# Sex, Type of Surgery, and Surgical Site Infections Are Associated with Perioperative Cortisol in Colorectal Cancer Patients

**DOI:** 10.3390/jcm10040589

**Published:** 2021-02-04

**Authors:** Mariusz G. Fleszar, Paulina Fortuna, Marek Zawadzki, Paweł Hodurek, Iwona Bednarz-Misa, Wojciech Witkiewicz, Małgorzata Krzystek-Korpacka

**Affiliations:** 1 Department of Medical Biochemistry, Wroclaw Medical University, 50-368 Wroclaw, Poland; mariusz.fleszar@umed.wroc.pl (M.G.F.); paulina.fortuna@umed.wroc.pl (P.F.); pawel.hodurek@umed.wroc.pl (P.H.); iwona.bednarz-misa@umed.wroc.pl (I.B.-M.); 2 Department of Oncological Surgery, Regional Specialist Hospital, 51-124 Wroclaw, Poland; zawadzki@wssk.wroc.pl (M.Z.); witkiewicz@wssk.wroc.pl (W.W.); 3 Department of Physiotherapy, Wroclaw Medical University, 51-618 Wroclaw, Poland; 4 Research and Development Centre at Regional Specialist Hospital, 51-124 Wroclaw, Poland

**Keywords:** metabolomics, mass spectrometry, hypothalamus–pituitary gland–adrenal glands axis, cortisol, stress response, surgical trauma, robotic surgery, minimal invasive surgery

## Abstract

Excessive endocrine response to trauma negatively affects patients’ well-being. Cortisol dynamics following robot-assisted colorectal surgery are unknown. We aimed at determining the impact of cancer pathology and surgery-related factors on baseline cortisol levels and analyzed its time-profile in colorectal cancer patients undergoing open or robot-assisted surgery. Cortisol levels were measured using liquid chromatography quadrupole time-of-flight mass spectrometry. Baseline cortisol was not associated with any patient- or disease-related factors. Post-surgery cortisol increased by 36% at 8 h and returned to baseline on postoperative day three. The cortisol time profile was significantly affected by surgery type, estimated blood loss, and length of surgery. Baseline-adjusted cortisol increase was greater in females at hour 8 and in both females and patients from open surgery group at hour 24. Solely in the open surgery group, cortisol dynamics paralleled changes in interleukin (IL)-1β, IL-10, IL-1ra, IL-7, IL-8 and tumor necrosis factor (TNF)-α but did not correlate with changes in IL-6 or interferon (IFN)-γ at any time-point. Cortisol co-examined with C-reactive protein was predictive of surgical site infections (SSI) with high accuracy. In conclusion, patient’s sex and surgery invasiveness affect cortisol dynamics. Surgery-induced elevation can be reduced by minimally invasive robot-assisted procedures. Cortisol and C-reactive protein as SSI biomarkers might be of value in the evaluation of safety of early discharge of patients.

## 1. Introduction

Cortisol, the primary glucocorticoid, is released in reaction to the activation of the hypothalamus–pituitary gland–adrenal glands (HPA) axis. Since cortisol has a profound effect on protein, carbohydrate, and lipid metabolism as well as on immune and inflammatory response [1,2], it is being tested as a candidate biomarker in diverse pathological states [3,4,5]. As a stress hormone, cortisol is essential for maintaining homeostasis following trauma by regulating the synthesis of catecholamines and adrenergic receptors and maintaining vascular tone [6].

While it is well known that cortisol concentration rapidly increases in the response to surgery-induced trauma, both its dynamics in the perioperative period and the relevant modifying factors are not clearly defined [7]. The vast majority of available data has been obtained from small and heterogeneous studies, in which cortisol had been quantified using immunoenzymatic assays [7]. Immunoassays are characterized by poor specificity and low repeatability [8] and exaggerate postoperative cortisol elevation [7]. In this study, we adapted methyl tert-butyl ether (MTBE) liquid–liquid extraction method [9,10] for cortisol quantification. We evaluated its dynamics in the perioperative period in patients with colorectal cancer undergoing open and robotic surgery using liquid chromatography quadrupole time-of-flight mass spectrometry (LC-QTOF-MS).

Colorectal cancer (CRC) is the third most common type of cancer and the second leading cause of cancer death globally. The highest rates of incidence occur in developed countries, as associations may be drawn between CRC and Western diets and lifestyles [11]. Surgical removal of the tumor and regional lymph nodes remain the mainstay of CRC therapy. For many years, this was performed through open surgery. Yet, over the last two decades, minimally invasive techniques have become the preferred approach of Western surgeons. The benefits of minimally invasive surgery include shorter hospital stay, earlier return to normal function, reduced postoperative pain, and others [12,13]. The most popular minimally invasive approach is laparoscopic surgery. However, it is lacking due to poor ergonomics, rigid fixed-tip instruments, and a physiological tremor. As a result, laparoscopy is associated with a steep learning curve and high conversion rates to open surgery, which happen when it is not possible to continue the operation laparoscopically [14]. Robotic surgical systems were introduced to overcome the limitations of laparoscopy. They are equipped with instruments with flexible tips and a jointed wrist, offering a movement range of seven degrees, stable and surgeon-controlled 3-D camera, tremor filtering, and improved overall ergonomics [15]. Since its introduction, robotic surgery has been widely accepted, with around 1.2 million cases of robotic surgery performed worldwide [16]. Still, data concerning body reaction to robot-assisted colorectal surgery are scarce and limited to inflammatory and immune response [17,18,19,20,21,22]. The potential impact of robot-assisted approach on cortisol secretion remains unknown. Therefore, the aims of this study were (1) quantifying cortisol changes in plasma of CRC patients in the perioperative period using LC-QTOF-MS in reference to immune and inflammatory cytokines; (2) identifying potential patient-, cancer-, and surgery-related factors, including the type of surgery, which affect the hormone concentration profile over time; and (3) determining the potential of cortisol as a predictor of adverse clinical outcomes.

## 2. Materials and Methods

### 2.1. Patients

This is a follow-up research conducted on biobanked samples collected for the purpose of a prospective, comparative, non-randomized study carried out as part of the Wrovasc–Integrated Cardiovascular Centre project (http://www.wssobr-wroc.pl/projekty/wrovasc/ (accessed on 18 November 2020)). The study population included 76 unselected patients who underwent curative resection for histologically confirmed colorectal adenocarcinoma in the Department of Surgical Oncology of the Regional Specialist Hospital in Wroclaw, Poland between 2012 and 2015. Exclusion criteria included age < 18 years, ASAPS > 3, emergency surgery, patients with gross metastatic disease, locally advanced cancers not amenable to curative resection, patients with tumors requiring en bloc multi-visceral resection, and the presence of coexisting malignancies. Patients receiving steroids and/or immunosuppressive agents were excluded as well.

Blood samples were collected in the morning prior to surgery (8:00) and at 8, 24, and 72 h post-incision. Surgeries were started at 9 am to avoid the confounding effect of diurnal variation in cortisol concentration. The basic characteristics of enrolled patients are given in Table 1. A complete set of samples from all time points was available for 61 patients, of whom 34 underwent open and 27 underwent robot-assisted surgery using the da Vinci^®^ Si surgical system (Intuitive Surgical, Sunnyvale, CA, USA) (Table 2). All patients were given a choice between open and robotic surgery. Advantages and disadvantages of both surgical options were discussed in detail with all patients. Following those discussions, patients chose their preferred surgical technique. Patient data, including patient’s demographics, comorbidities, perioperative complications, and pathology report, were collected prospectively. The general health status of enrolled patients was assessed using the Charlson Comorbidity Score (CCS) and the American Society of Anesthesiologists Physical Status Classification System (ASAPS).

Patients underwent standardized general anesthesia. Intravenous agents such as propofol, fentanyl, and rocuronium were used for induction. Anesthesia was maintained with sevoflurane. Local or epidural anesthesia was not applied in the study group. All patients were given NSAIDs (metamisol) before waking up or immediately after the surgery in the recovery room.

Postoperative complications were recorded within 30 days after surgery and classified in accordance with the Clavien–Dindo Classification (CDC). Surgical site infections (SSI), present in 15 (24.6%) patients, were recorded prospectively for all patients and classified in accordance with the Center for Disease Control and Prevention criteria. Restoration of bowel function (RoBF) was defined as tolerance of solid diet and the passage of first stool. A ≥5 day cut-off was used to define prolonged (pathological) postoperative ileus [23], which was present in 24 (39%) patients.

### 2.2. Analytical Methods

#### 2.2.1. Plasma

Blood samples were collected by venipuncture using citrate as an anticoagulant. Plasma was separated from cells by sample centrifugation at 2000× *g* for 15 min at 10 °C, aliquoted, and stored at −80 °C until investigation.

#### 2.2.2. Chemicals

Cortisol and Cortisol-D4 were obtained from Sigma-Aldrich (Poznan, Poland). Methanol, methyl tert-butyl ether, water, and formic acid were acquired from Merck Millipore (Warsaw, Poland), and leucine–enkephalin was obtained from Waters (Warsaw, Poland).

#### 2.2.3. LC-QTOF-MS

Plasma total cortisol was measured by the LC-QTOF-MS method, developed, and validated by our group, which was devised using a well-described liquid–liquid extraction approach with methyl tert-butyl ether (MTBE) as extraction reagent [9,10].

Calibration standards and plasma samples were prepared according to the same protocol. Briefly, 50 µL aliquots of the calibration standards (in the range from 25 to 500 ng/mL) of human plasma were mixed with 10 µL of internal standard solution (1 µg/mL of D4-cortisol in methanol) and vortexed (1 min, 25 °C). The samples were extracted with 400 µL of MTBE at 25 °C for 10 min. Then, the mixture was centrifuged at 12,500 RPM for 5 min at 4 °C. Supernatants were dried in vacuum evaporator at 50 °C for 30 min and the resulting lyophilizates were re-dissolved in 25% methanol in water. A detailed description of the method along with results of its validation are presented in the Supplementary Materials (Table S1).

#### 2.2.4. Immunoassays

Previously published data [18,19,20] on immune and inflammatory interleukins were retrieved from our database for correlation analysis. Interleukin concentration was determined in serum samples, using a flow cytometry-based method (Luminex xMAP^®^ technology), which utilizes magnetic beads conjugated with specific monoclonal antibodies, using the Bio-Plex 200 platform (Bio-Rad, Hercules, CA, USA) and Bio-Plex Pro™ Human Cytokine, Chemokine, and Growth Factor Magnetic Bead-Based Assays. Data on interleukins were available for all patients included in the current study at all indicated time-points (preoperative and 8 h, 48 h, and 72 h post-incision).

#### 2.2.5. Laboratory Parameters

Data on lymphocyte (LYMPH), neutrophil (NEU), and leukocyte (WBC) counts, which were obtained using an automated hematology analyzer, were retrieved from patient’s medical history. They were determined preoperatively and 24 h post-incision. The concentrations of high-sensitive C-reactive protein (CRP) were measured on the Architect 4100 Ci analyzer (Abbott Laboratories, Lake Bluff, IL, USA) using dedicated Multigent CRP Vario immunoturbidimetric tests from Abbott Laboratories. They were available for 58 of patients at all indicated time-points (preoperative and 8, 48, and 72 h post-incision).

### 2.3. Statistical Analysis

The Kolmogorov–Smirnov test was used to analyze data normality, and the Levene test was used to analyze homogeneity of variances. Data on cortisol were non-normally distributed and/or had non-homogeneous variances and therefore were log-transformed prior to analysis. Data were examined using t-test for independent samples (with Welch correction for unequal variances) or one-way analysis of variance (ANOVA) with Student–Newman–Keuls *post-hoc* test and presented as means with standard deviation (SD) or 95% confidence interval (CI). Non-normally distributed data or with no homogeneity of variances were analyzed using the Mann–Whitney U test or Kruskal–Wallis *H* test with Conover *post-hoc* test and presented as medians with 95% CI or interquartile range (IQR). Cortisol dynamics were analyzed using repeated measures ANOVA. The least squares multiple regression (stepwise method) was used to establish independent associations. Variables were entered into the model if *p <* 0.05 and removed if *p* > 0.1. Partial correlation coefficients (r_p_) with the effect of the co-variables removed were calculated. Data frequency was compared using Fisher’s exact test or χ^2^ test. Correlation analysis was conducted using Pearson correlation (r) or Spearman rank correlation (ρ). The receiver operating characteristics (ROC) curve analysis was conducted to assess the predictive power of cortisol as an SSI marker. The area under ROC curve (AUC) expressed in percentage was indicative of its overall accuracy. Sensitivity and specificity were calculated for an optimal cut-off as well. Logistic regression (enter method) with SSI as explained and cortisol and CRP as explanatory variables was used to calculate predicted probabilities to be used as a dependent variable in ROC analysis. Linear least squares regression with coefficient of determination (R^2^) indicative of the goodness of fit, Snedecor *F* test, and *t*-test for independent samples were used to validate the LC-QTOF-MS method of cortisol determination.

To discern factors affecting cortisol dynamics, data on body mass index (BMI) were dichotomized using World Health Organization (WHO) criteria for overweight/obesity as a threshold (≥25 kg/m^2^). In view of lack of objective cut-offs for other variables, arbitrary thresholds based on medians in the whole cohort (150 mL for estimated blood loss (EBL), 15 for number of excised lymph nodes, 175 min and 6 days for length of surgery and hospital stay, respectively) or previously published cut-offs (75 yrs. for age [24,25]) were applied for data dichotomization [26].

All calculated probabilities were two-tailed. *p* values ≤ 0.05 were considered statistically significant. The analyses were conducted using MedCalc Statistical Software version 19.3.1 (MedCalc Software Ltd., Ostend, Belgium; https://www.medcalc.org; 2020).

## 3. Results

### 3.1. Preoperative Cortisol

None of the examined patient-related factors, which were, age, sex, body mass index (BMI), general health condition assessed in terms of ASAPS or CCS, had a significant effect on baseline cortisol concentration. Likewise, cancer pathology had no significant effect (Supplementary Material, Table S2).

### 3.2. Postoperative Cortisol

**Figure 1:**
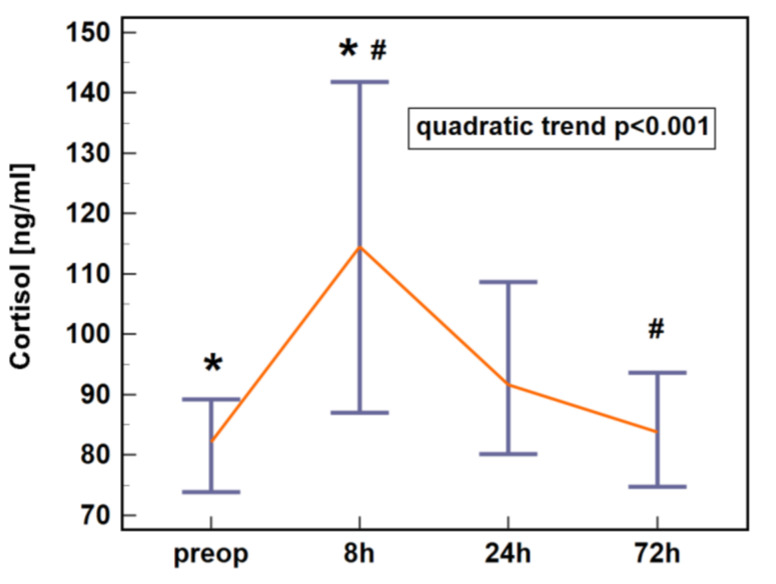
Plasma cortisol dynamics in early postoperative period following colorectal surgery. Data are presented as medians with 95% CI. Data were analyzed following log-transformation using repeated measures analysis of variance (*n* = 61). Significant differences between time points are indicated by symbols of the same type (*, #). CI, confidence interval; preop, preoperative; 8 h, 8 h post-incision; 24 h, 24 h post-incision; 72 h, 72 h post-incision.

Cortisol concentration in early postoperative period following colorectal surgery displayed a quadratic trend. It increased at 8 h post-incision and dropped at 24 and 72 h (Figure 1).

#### 3.2.1. Patient-Related Modifiers

**Figure 2:**
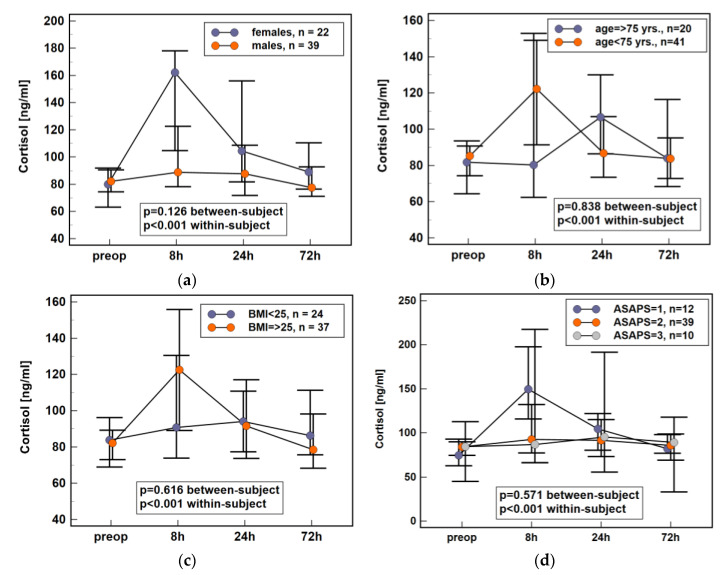
Impact of various patient characteristics on plasma cortisol dynamics in early postoperative period following colorectal surgery: (**a**) sex; (**b**) age; (**c**) body mass; (**d**) health status. Data presented as medians with 95% CI. Data were analyzed following log-transformation using repeated measures analysis of variance with p values indicating significance of between-subjects (sex, age, body mass, and ASAPS) and within-subjects (time points) effects (*n* = 61). BMI, body mass index; yrs., years; ASAPS, the American Society of Anesthesiologists physical status classification system; CI, confidence interval; preop, preoperative; 8 h, 8 h post-incision; 24 h, 24 h post-incision; 72 h, 72 h post-incision.

Repeated-measures ANOVA was used to assess the potential effect of patient-related factors such as sex, age, body mass, and general health condition (American Society of Anesthesiologists Physical Status Classification System, ASAPS). For the purpose of analysis, age and body mass were dichotomized using 75 yrs. and BMI ≥ 25 as cut-offs. As between-subject factors, none of the above had significant impact on plasma cortisol dynamics following colorectal surgery. Although the effect of sex did not reach statistical significance, female patients tended to have a more marked elevation (Figure 2). Therefore, the relative change in cortisol concentration between 8 h post-incision and baseline (measurement at 8 h/measurement at baseline; Δ8/0) as well as between 24 h post-incision and baseline (measurement at 24 h/measurement at baseline; Δ24/0) was calculated and compared. Both Δ8/0 and Δ24/0 were significantly higher in females than males (Δ8/0: 1.79 (95%CI: 1.3–2.4) vs. 1.19 (1–1.5), *p* = 0.022 and Δ24/0: 1.54 (1.2–2) vs. 1.0 (0.9–1.2), *p* = 0.009). Individual cortisol concentrations are provided in Supplementary Materials Table S3.

#### 3.2.2. Surgery-Related Modifiers

**Figure 3:**
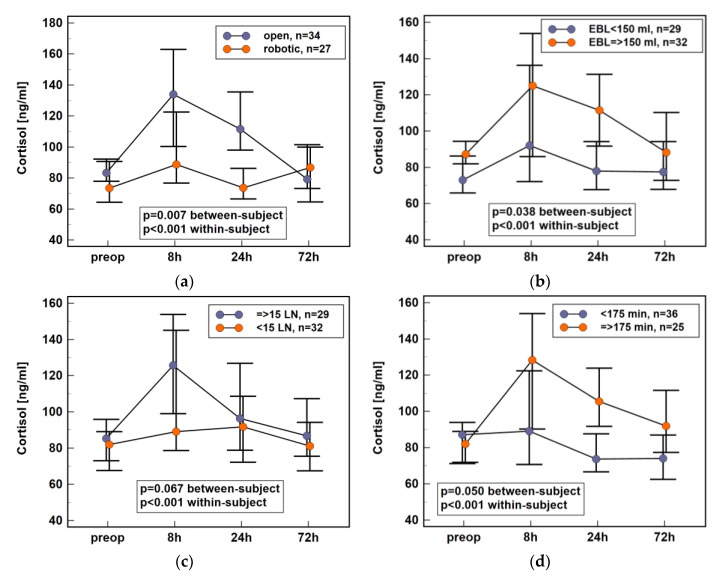
Impact of surgery-related data on plasma cortisol dynamics in early postoperative period following colorectal surgery: (**a**) surgery type; (**b**) estimated blood loss; (**c**) number of harvested lymph nodes; (**d**) length of surgery. Data presented as medians with 95% CI. Data were analyzed following log-transformation using repeated measures analysis of variance with *p* values indicating significance of between-subjects (surgery type, extent, length, and EBL) and within-subjects (time points) effects (*n* = 61). EBL, estimated blood loss; LN, lymph nodes; CI, confidence interval; preop, preoperative; 8 h, 8 h post-incision; 24 h, 24 h post-incision; 72 h, 72 h post-incision.

Surgery-related factors such as surgery type (open vs. robotic), number of harvested lymph nodes (using ≥15 as a cut-off), estimated blood loss (EBL) (using ≥150 mL as a cut-off), and length of surgery (using ≥175 min as a cut-off) were analyzed. Dynamics of cortisol concentration in early postoperative period differed significantly between patients undergoing open and robot-assisted colorectal surgery (Figure 3a), between patients with non-substantial and substantial blood loss (Figure 3b), and with respect to surgery length (Figure 3c). It also tended to differ with respect to the extent of the procedure, which was expressed by the number of harvested lymph nodes, with cortisol levels appearing lower in patients with less than 15 lymph nodes excised (Figure 3d). Individual cortisol concentrations are provided in Supplementary Materials Table S4.

#### 3.2.3. Independent Modifiers (Multiple Regression)

Type of surgery is closely related to EBL and length of surgery (Table 2). Therefore, to discern independent modifiers of cortisol time profile, the effects of those surgery-related factors and sex were co-analyzed in the least squares multiple regression. The increase after 8 h (Δ8/0) was independently associated with sex, being higher in females (partial correlation coefficient r*_p_* = 0.29, *p* = 0.022). The change after 24 h in relation to baseline (Δ24/0) was independently associated with sex (r*_p_* = 0.27, *p* = 0.038) and surgery type (r*_p_* = 0.29, *p* = 0.026), being higher in females and patients undergoing open surgery. The change after 72 h post-incision (Δ72/0) was independently associated with length of surgery (r*_p_* = −0.31, *p* = 0.015), dropping in patients with prolonged surgery.

### 3.3. Cortisol and Inflammatory/Immune Response

Cortisol is involved in bidirectional relationships with cytokines representing Th1 (interferon (IFN)-γ) and Th2 (interleukin (IL)-4 and IL-10) immune responses as well as with classic pro-inflammatory cytokines (IL-1β, tumor necrosis factor (TNF-α, IL-6, and IL-8) and immunomodulatory IL-7. Therefore, we examined whether changes in cortisol during the perioperative period were associated with changes in leukocyte counts and cytokine dynamics.

Relative change in cortisol after 8 h post-incision as compared to baseline (Δ8/0) correlated positively with respective change in IL-10 (r = 0.38, *p* = 0.022) and IL-7 (r = 0.34, *p* = 0.040) but exclusively in patients undergoing open surgery. No correlations with changes in IL-1β, IL-1ra, IL-4, IL-6, IL-8, TNFα, and IFNγ were observed.

Relative change in cortisol after 24 h post-incision as compared to baseline (Δ24/0) correlated positively with respective change in IL-1β (r = 0.34, *p* = 0.049), IL-1ra (r = 0.38, *p* = 0.027), and IL-8 (r = 0.38, *p* = 0.027) and tended to correlate with IL-10 (r = 0.32, *p* = 0.066) and TNFα (r = 0.32, *p* = 0.064) but exclusively in patients undergoing open surgery. No correlations with changes in IL-4, IL-6, IL-7, and IFNγ were observed.

Relative change in cortisol after 72 h post-incision as compared to baseline (Δ72/0) correlated positively with respective change in IL-1β (r = 0.38, *p* = 0.029), IL-10 (r = 0.36, *p* = 0.040), and TNFα (r = 0.34, *p* = 0.049) and tended to correlate with IL-1ra (r = 0.34, *p* = 0.052) but exclusively in patients undergoing open surgery. No correlations with changes in IL-4, IL-6, IL-7, IL-8, and IFNγ were observed.

No correlation with white blood cells (WBC), lymphocyte (LYMPH), or neutrophil (NEU) counts was observed.

### 3.4. Surgical Adverse Events

There was no association between cortisol concentration at 24 and 72 h adjusted to baseline (Δ24/0 and Δ72/0) and complication rates following colorectal surgery, which was expressed as CDC and dichotomized using ≥3 cut-off. The potential association with specific surgical adverse events such as anastomotic leak (AL), delayed restoration of bowel function (RoBF), surgical site infections (SSI), or prolonged hospitalization was examined as well. The increase of baseline-adjusted cortisol at 24 h (Δ24/0) was significantly higher in patients with SSI than without (1.54 (95%CI: 1.1–2.1) vs. 1.12 (1–1.3), *p* = 0.049). Likewise, Δ72/0 was significantly higher in patients with SSI than without (1.25 (95%CI: 1.1–1.5) vs. 0.93 (0.8–1.1), *p* = 0.043). No association with AL, RoBF, or hospital stay was found.

### 3.5. Cortisol as SSI Marker

**Figure 4:**
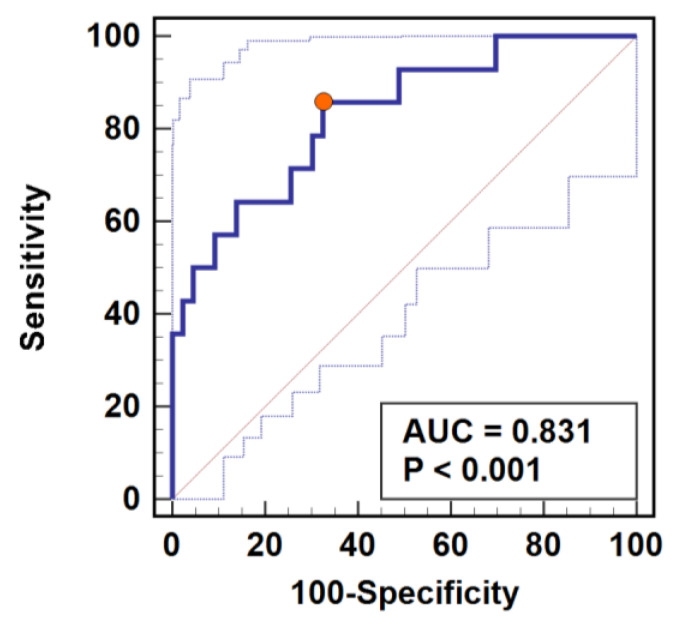
Predictive power of combined assessment of cortisol and C-reactive protein (CRP) as surgical site infection (SSI) marker. Determination of cortisol change at 24 h post-incision adjusted to baseline concentration (Δ24/0) was combined with CRP quantification at 72 h post-incision. Data presented as receiver operating characteristics (ROC) curve. AUC, area under ROC curve representative of marker accuracy with 95% confidence interval. Closed orange circle indicates an optimal cut-off corresponding with the highest combined sensitivity and specificity.

We compared the power of cortisol and C-reactive protein (CRP) to predict SSI prior to their clinical manifestation. Cortisol was evaluated as a change at 24 h or 72 h in relation to baseline concentration (Δ24 h/0 or Δ72 h/0 ratios) or as absolute values measured at 24 or 72 h. Cortisol concentration adjusted to baseline was superior to absolute hormone concentration at 24 or 72 h. Change in cortisol at 24 h (Δ24 h/0 ratio) and CRP concentration at 72 h were superior individual predictors of SSI. Cortisol as SSI biomarker was characterized by high sensitivity but low specificity, while CRP at 72 h was characterized by low sensitivity but high specificity (Table 3). Combined evaluation of both—cortisol Δ24 h/0 and CRP at 72 h—had improved overall accuracy (83%) and high sensitivity (86%) accompanied by moderate specificity (67%) (Table 3, Figure 4).

## 4. Discussion

Surgery causes increased cortisol output. Both overly elevated and insufficient cortisol elevation are disadvantageous, the former impeding wound healing and the latter inducing an adrenal crisis, which is life-threatening. However, the magnitude of cortisol elevation as well as affecting factors remain to be precisely determined [7]. The available data are derived mostly from small and highly heterogeneous cohorts with meta-data collected from patients undergoing surgery for diverse conditions. Moreover, cortisol has been quantified using immunoassays, cross-reacting with endo- and exogenous steroids and thus exaggerating its upregulation. In the present study, cortisol was quantified using mass-spectrometry and in a comparatively large cohort, it is homogeneous with respect to patients’ ethnicity (Caucasian), underlying condition (CRC amenable for curative resection), and applied anesthesia. Patients were operated using open or robot-assisted technique, allowing us to investigate the effect of minimally invasive approach on cortisol dynamics within one study.

We showed that cortisol concentration increased only by 36% at 8 h post-incision and returned to baseline on postoperative day three. Postoperative cortisol dynamics were affected mostly by surgery-related factors, predominantly by the invasiveness of surgical technique. In addition, they reflected the degree of inflammatory response and were predictive of surgical site infections. A better understanding of cortisol time profile and its modifiers is of high clinical relevance. It may help tailor glucocorticoid replacement therapy and prevent stress-induced adrenal crisis in patients with adrenal insufficiency (AI) [7,27]. Central AI is a likely consequence of chronic glucocorticoid therapy used in patients with inflammatory bowel disease [28], which itself is a condition associated with increased risk of CRC development. In turn, cortisol elevation and its disturbed diurnal and/or nocturnal rhythm are associated with cancer and old age [29,30]. As life expectancy increases, the demographic structure of CRC patients changes. Surgery in seniors bears higher risk of complications and mortality [31]. Although adrenal aging might contribute to the phenomenon [32], neither older age nor health condition, expressed in terms of ASAPS or CCS, had a significant impact on cortisol dynamics. In addition, there was no correlation between the hormone and the Clavien–Dindo Classification of postoperative complications. Lack of cortisol association with age might result from the hormone being determined in the morning as age-related differences are rather evidenced in the late evening and early night [33].

It has been repeatedly shown that excessive cortisol elevation during trauma is disadvantageous, as it skews the immunomodulatory properties of cortisol toward anti-inflammatory and immunosuppressive [34]. Cortisol reduces leukocyte number, promotes suppressor subpopulations of lymphocytes, and interferes with immune cell migration and interleukin production [34]. A suppressed immune system enables cancer and may facilitate its recurrence [35,36,37]. It also hinders the healing process of surgical wounds and interferes with immune response to pathogen [38]. Accordingly, we noted higher baseline-adjusted cortisol concentrations on the 1st and 3rd postoperative day in patients who subsequently developed SSI. Since predictive biomarkers for perioperative complications following colorectal resection are sought after [39], we evaluated cortisol as a potential SSI marker and compared it to CRP. The SSI is easily diagnosed clinically, and therefore, there was no clear need to investigate its predictive markers in the past. However, SSI typically occurs on the 5–7th postoperative day. Yet, since the introduction of the so-called “fast track” Enhanced Recovery After Surgery (ERAS) perioperative care protocol [40], colorectal surgeons are able to discharge patients home as early as the 3rd postoperative day. This scenario carries the risk of developing full clinical signs of SSI at home, where access to medical expertise is limited. Establishing reliable markers of SSI would permit early discharge without risk of serious complications. Our results showed that cortisol was superior to CRP at 24 h, while CRP was better SSI marker at 72 h. However, cortisol displayed only a moderate accuracy, albeit with superior sensitivity but not specificity. CRP at 72 h as an SSI marker displayed superior specificity over sensitivity. Therefore, we tested the predictive power of combined quantification of both markers. We found that cortisol ratio at 24 h as compared to baseline (>1.1) combined with CRP at 72 h (>195 mg/L) is an accurate predictor of SSI, which is characterized by high sensitivity and moderate specificity.

As already mentioned, glucocorticoids interfere with leukocyte number, their migration, and cytokine production. For example, they promote immunosuppressive Th2 cytokines (e.g., IL-10 and IL-4) at the expense of pro-inflammatory Th1 cytokines (e.g., IFNγ) [34]. Therefore, cortisol may underline and/or aggravate Th2 dominance induced by cancer and surgery, compromising patient’s immunocompetence against the disease and pathogens. Consequently, cortisol and Th1/Th2 imbalance might contribute to cancer recurrence and increased susceptibility to infections in the postoperative period [41]. Corroborating the notion on hormone interplay with Th1/Th2 cytokines, changes in cortisol concentration positively correlated with changes in IL-10, although there was no observed association with leukocyte counts or IFNγ, which is a Th1 representative. In line with hormone synthesis being stimulated by classic pro-inflammatory cytokines, cortisol correlated positively with IL-1β, TNFα, and IL-8 but not with secondary inflammatory mediators such as IL-6. Interestingly, the initial cortisol elevation also correlated directly with IL-7, which is an intriguing pleiotropic cytokine with known immunomodulatory function [42], increasingly acknowledged pro-tumorigenic activity [43,44,45], and a currently investigated role in energy homeostasis and metabolism [46,47]. The upregulation of IL-1β is accompanied by an increase in its receptor agonist (IL-1ra) to prevent hyperinflammation. Consistently, perioperative changes in cortisol were correlated positively with anti-inflammatory IL-1ra as well. Remarkably, cortisol association with cytokines was evident exclusively in the open surgery group. It probably results from greater perioperative dynamic range after open than robotic surgery in both cortisol, as demonstrated in present study, and in cytokines, as shown in our earlier work [18,19,20]. Indeed, the extent of trauma has been repeatedly reported to affect cortisol [13]. According to Johns Hopkins surgical criteria, resections of the digestive tract are considered moderately invasive (grade II). Within this grade, we were able to demonstrate that robot-assisted colorectal surgery elicited less marked cortisol elevation than a more invasive open procedure. Similar observations have been made for laparoscopic and open cholecystectomy [48], robotic radical prostatectomy and open retropubic approach [49], and for robotic and open hysterectomy [50].

Pain is believed to trigger stress hormone secretion proportionally to its severity [51]. Therefore, minimally invasive procedures might elicit flattened cortisol response because of reduced pain in addition to diminished inflammatory response. Indeed, laparoscopic colorectal surgery has been associated with a less postoperative pain as compared to open procedure [52]. Correspondingly, our patients after robotic operations, in whom cortisol concentrations are lower, experienced less pain (self-reported), which appeared to be reflected in a lower demand for painkillers. Still, a link between the degree of self-reported pain and cortisol elevation has recently been questioned [53].

Anesthetic procedures are important modifiers of perioperative cortisol dynamics [7,54]. General anesthesia was induced in our patients with propofol, which does not inhibit cortisol synthesis in therapeutic doses [55]; rocuronium, which demonstrates no effect [56]; and fentanyl. Similar to other opioids, fentanyl is a known suppressor of the HPA axis [54], and its chronic application induces AI, but the short-term effects on steroidogenesis in humans are not known [57]. Nonetheless, all our patients were subjected to the same anesthetic protocol; therefore, the potential interference was minimized. Anesthesia was maintained with sevoflurane, which has been shown to reduce postoperative cortisol peak by two-fold as compared to isoflurane [58]. Considering the inhibitory effect of sevoflurane and the fact that robotic procedures lasted significantly longer, it might potentially contribute to the lower cortisol peak in this group. However, the length of surgery had no significant impact on the initial peak. Rather, it affected cortisol normalization, which was faster in patients who were on operated longer. Still, with the effects of other modifiers accounted for, the length of surgery was an independent predictor of cortisol on 3rd postoperative day; thus, it was not likely a direct effect of sevoflurane. Our observation corroborates the results of the meta-analysis, in which during grade II surgical procedures, initial cortisol was lower following shorter operations. However, starting with postoperative day one, shorter surgeries were accompanied with higher cortisol. Still, the impact of surgery duration within 48 h has been deemed non-significant [7].

Apart from surgery type, the estimated blood loss and number of harvested lymph nodes also affected cortisol dynamics. However, only the surgical approach was an independent predictor of a change in its concentration after 24 h. Other studies have shown that cortisol response might be stronger in females [7]. While there were no sex-related differences in baseline cortisol, female patients had significantly higher baseline-adjusted increases at 8 and 24 h post-incision. Moreover, the female sex was an independent predictor of cortisol elevation at those time-points.

Our study has several limitations that should be mentioned. As exploratory research conducted as a follow-up to another project, it is based entirely on previously collected and biobanked material, which made the earlier estimation of adequate sample size impossible. Nonetheless, the power analysis indicated that the study was sufficiently powered in case of biomarker analysis (>80%) and only slightly underpowered in case of cortisol being affected by surgery type (77% instead of 80%). Robotic surgery was a new technique in Poland at that time, so the choice of surgical approach was left to the patient. Consequently, the study is not randomized and relatively small-scale, which affects the analysis of the subgroups in particular. In case of cortisol evaluation, it would be optimal to investigate normal adrenal function before surgery and that cortisol is determined along with other hormones, such as ACTH or growth hormone, which are preferably quantified with higher frequency.

## 5. Conclusions

In the study presented here, an LC-MS/MS method for cortisol quantification in plasma was developed and validated. It allowed us to determine and map the changes in cortisol concentration over the perioperative period in patients with colorectal cancer, who were undergoing curative tumor resection. The goal was to establish the factors that modify those shifts. Female sex along with open surgery were responsible for higher baseline-adjusted cortisol concentrations at 8- and 24-h post-incision, respectively. Concomitant evaluation of baseline-adjusted cortisol at 24 and CRP at 72 h could help predict surgical site infections before their clinical manifestation with high accuracy. These findings, if validated in an independent and larger cohort, might make the early discharge of patients safer.

## Figures and Tables

**Table 1:** Characteristics of study population (baseline cortisol quantification).

Characteristics of Study Population	
*N*	76
Demographics:	
Sex, F/M (*n*)	30/46
Age, mean ± SD	67.5 ± 10.8
BMI, median (IQR)	26.6 (23.6;30.4)
ASAPS, 1/2/3 (*n*)	17/48/11
CCS, median (IQR)	5 (4–5)
Oncological features:	
TNM, 0/I/II/III/IV (*n*)	5/6/29/31/5
T, Tis/1/2/3/4 (*n*)	5/1/9/48/13
N, 0/1/2 (*n*)	40/20/16
M, 0/1 (*n*)	71/5
G, 1/2/3/4/x (*n*)	9/52/10/1/4
Anatomical site, RC/LC/R (*n*)	22/21/33
Surgical features:	
Surgery type, open/robotic (*n*)	39/37
Procedure, APR/LH/LAR/RH/SR (*n*)	5/4/28/22/17

*N*, number of observations; F, females; M, males; SD, standard deviation; BMI, body mass index; IQR; interquartile range; ASAPS, the American Society of Anesthesiologists Physical Status Classification System; CCS, the Charlson Comorbidity Score; TNM, tumor-node-metastasis staging system; G, histopathological grade; RC, right colon; LC, left colon; R, rectum; APR, abdominal perineal resection; LH, left hemicolectomy; LAR, low anterior resection; RH, right hemicolectomy; SR, sigmoid resection.

**Table 2:** Characteristics of study population (follow-up).

Parameter	Open Surgery	Robotic Surgery	*p* Value
*N*	34	27	
Demographics:			
Sex, F/M (*n*)	16/18	6/21	0.062
Age [yrs.], mean ± SD	69.6 ± 9.0	64.4 ± 13.7	0.082
BMI [kg/m^2^], median (IQR)	26.3 (23.6;29.1)	25.4 (23.6;28.4)	0.557
ASAPS, 1/2/3 (*n*)	8/21/5	4/18/5	0.680
CCS, median (range)	5 (4–8)	4 (3–7)	0.470
Oncological features:			
TNM, 0/I/II/III/IV (*n*)	2/2/14/13/3	3/2/11/9/2	0.952
T, Tis/1/2/3/4 (*n*)	2/1/2/22/7	3/0/4/15/5	0.599
N, 0/1/2 (*n*)	18/8/8	16/6/5	0.862
M, 0/1 (*n*)	31/3	25/2	1.0
G, 1/2/3/4/x (*n*)	3/25/4/1/1	5/14/5/0/3	0.337
Anatomical site, RC/LC/R (*n*)	6/13/15	9/8/10	0.365
Surgical features:			
Procedure, APR/LH/LAR/RH/SR (*n*)	1/1/14/6/12	1/3/9/9/5	0.305
Length of surgery [min.], mean ± SD	134.4 ± 52.8	213.1 ± 65.6	<0.001
EBL [mL], median (range)	200 (30–300)	100 (50–200)	<0.001
Harvested LN, median (range)	14.5 (12–17)	14 (12–19)	0.693
CDC [≥3], no/yes (*n*)	29/5	26/1	0.214
SSI, no/yes (*n*)	22/12	24/3	0.038
RoBF [days], median (range)	4 (2–7)	3.5 (2–9)	0.595
LoHS [days], median (range)	6 (4–20)	6 (3–8)	0.063

*N*, number of observations; F, females; M, males; SD, standard deviation; BMI, body mass index; IQR; interquartile range; ASAPS, the American Society of Anesthesiologists Physical Status Classification System; CCS, the Charlson Comorbidity Score; TNM, tumor-node-metastasis staging system; G, histopathological grade; RC, right colon; LC, left colon; R, rectum; APR, abdominal perineal resection; LH, left hemicolectomy; LAR, low anterior resection; RH, right hemicolectomy; SR, sigmoid resection; EBL, estimated blood loss; LN, lymph nodes; CDC, the Clavien–Dindo Classification of postoperative complications; SSI, surgical site infections; RoBF, restoration of bowel function; LoHS, length of hospital stay.

**Table 3:** Cortisol and C-reactive protein (CRP) as markers of surgical site infections (SSI).

Marker	AUC (95% CI), *p*	Cut-Off	Sens. and Spec.
Cortisol at 24 h	0.692 (0.56–0.80), *p* = 0.023	>96.3 ng/mL	80 and 63%
Cortisol, Δ24 h/0	0.738 (0.61–0.84), *p* = 0.002	>1.08 ratio	93.3 and 54.4%
CRP at 24 h	0.693 (0.56–0.81), *p* = 020	>125.3 mg/L	73.3 and 72.1%
Panel I ^1^	0.788 (0.66–0.88), *p* < 0.001	>0.349 ^4^	73.3 and 83.7%
Cortisol at 72 h	0.664 (0.53–0.78), *p* = 0.046	>109.8 ng/mL	53.3 and 84.8%
Cortisol, Δ72 h/0	0.693 (0.560–0.81), *p* = 0.006	>0.9 ratio	93.3 and 47.8%
CRP at 72 h	0.746 (0.61–0.85), *p* = 0.004	>195 mg/L	50 and 95.4%
Panel II ^2^	0.774 (0.64–0.87), *p* < 0.001	>0.115 ^4^	100 and 41.9%
Panel III ^3^	0.831 (0.71–0.92), *p* < 0.001	>0.169 ^4^	85.7 and 67.4%

AUC, area under receiver operating characteristics (ROC) curve, indicative of marker’s overall accuracy; CI, confidence interval; Sens., sensitivity; spec., specificity; CRP, C-reactive protein. ^1^ panel consisting of ratio of cortisol at 24 h post-incision to its baseline concentration (cortisol Δ24 h/0) and CRP measured at 24 h; ^2^ panel consisting of ratio of cortisol at 72 h post-incision to its baseline concentration (cortisol Δ72 h/0) and CRP measured at 72 h; ^3^ panel consisting of ratio of cortisol at 24 h post-incision to its baseline concentration (cortisol Δ24 h/0) and CRP measured at 72 h; ^4^ predicted probabilities calculated in logistic regression analysis.

## Data Availability

Data sharing is not applicable to this article.

## Supplementary Materials

The following are available online at https://www.mdpi.com/2077-0383/10/4/589/s1, Details on LC-MS/MS analysis, Table S1: Results of method validation, Table S2: Baseline plasma cortisol association with patient- and cancer-related features, Table S3: Impact of patient-related features on mean cortisol concentration during perioperative period; Table S4: Impact of surgery-related features on mean cortisol concentration during perioperative period.
