# Supplementary material for: Sex, Type of Surgery, and Surgical Site Infections Are Associated with Perioperative Cortisol in Colorectal Cancer Patients
Source: J Clin Med. 2021 Feb 4;10(4):589. doi: 10.3390/jcm10040589 (PMC7914878; doi:10.3390/jcm10040589)
Supplement: Supplementary file 1 [file jcm-10-00589-s001.pdf]

Journal of Clinical Medicine

MDPI

Supplementary material for Fleszar et al.

1. Details on LC-MS/MS analysis

Chromatography

Chromatographic separation was conducted on nanoAcquity HSS T3 column (C18-phase, internal diameter 1 mm, length 50 mm, particle size 1.75 μm) using nanoAcquity UPLC system (Waters). Column was heated to 40°C. Sample injection volume was 2 μL. Total run time of the method was 9.5 min with flow rate of 120 μL/min. The 0.1% FA in water was used as a mobile phase A and 0.1% FA in methanol as a mobile phase B. The linear gradient was as follows: 25% B for 0.7 minute, from 25% to 90% B in 3.3 minute, from 90% to 95% B in 1 minute, 95% B for 1.5 minute and from 95% to 25 % B in 0.10 minute; re-equilibration time was 1.99 minute.

Mass spectrometry

Mass spectra were acquired using Xevo G2 QTOF MS (Waters) with electrospray ionization source (ESI) in a positive ionization mode. Source parameters were as follow: 2.5 kV (spray voltage), 120°C (source temperature) and 350°C (desolvation temperature). Nitrogen was used as the nebulizing (600 L/h) and drying gas (30 L/h). Data acquisition was carried on MassLynx software (Waters) in sensitivity mode with scan range from 275 to 560 m/z, using extracted ion chromatograms for quantitative analysis, following ions with mass accuracy of 0.02 Da were used: m/z 363.2166 and m/z 367.2418 for cortisol and cortisol-D4, respectively.

Method validation

The method was validated for linearity, accuracy, precision, and recovery, and limits of detection (LOD) and quantification (LOQ) were established.

Linear regression analysis of relative areas responses vs. concentration was conducted. The range of calibration curve was determined by the assessment of homogeneity of variances of the lowest and highest calibration levels using Snedecor F test. The calculated F value (F_{cal}=the highest relative standard deviation (RSD) for calibration level/the lowest RSD for calibration level) was 2.2 and for n=15 and α=0.05 was below the expected F value (F_{crit}=2.44), indicative of the correctness of calibration curve range. Calibration curve linearity was determined by coefficient of determination (R^2=0.996; acceptable threshold ≥0.995) and t-test for slope significance. The calculated t value (t_{cal}=slope/slope SD) was 102.23 and for n=120 and α=0.05 was above the expected t value (t_{cri}=1.98), indicative of excellent calibration curve linearity.

Accuracy and precision were determined for quality control samples at low, medium, and high concentrations: QC1=0.035 μg/mL, QC2=0.150 μg/mL, and QC3=0.450 μg/mL. The between-run accuracy and precision ranged from 98.6% to 112.3% and from 3.0% to 8.6%, respectively. Results are summarized in Table 3.

Recovery rates were determined using pooled plasma samples spiked with cortisol at the concentration of 0.039, 0.071 and 0.120 μg/mL. Respective mean recovery rates were as follows: 101.8%, 98.4%, and 94.8% (Table S1).

The LOD and LOQ were defined by a signal-to-noise (S/N) ratio, obtained for the lowest standard of the calibration curve. The S/N ratios were performed by comparing measured signals from samples with known low concentrations of analyte with those of blank samples. All calculations were conducted using MassLynx software (Waters) and Peak-to-Peak values of S/N were chosen for estimating LOD and LOQ. The LOD and LOQ values were 0.008 μg/mL (S/N=3:1) and 0.025 μg/mL (S/N=10:1), respectively.

Table S1. Results of method validation

	Sensitivity		Linearity		Precision and accuracy						
					Within-run (n = 8)				Between-run (n = 16)		
**Compound**	**LOD [μM]**	**LQQ [μM]**	R^2	**Slope tcal>tcri**	**Expected conc. [μM]**	**Measured conc. [μM]**	**Precision (CV%)**	**Accuracy [%]**	**Measured conc. [μM]**	**Precision (CV%)**	**Accuracy [%]**
					0.097	0.103±0.010	9.9	105.8	0.108±0.011	8.6	112.3
Cortisol	0.022	0.069	0.996	102.23>1.98	0.041	0.39±0.023	6.0	95.1	0.408±0.044	5.2	98.6
					1.24	1.31±0.024	1.8	105.5	1.34±0.044	3.0	107.8

	Mean recovery rates of the method				Precision for spiked matrix	
	**Plasma Measured conc. [μM] mean ± SD**	**Added conc. [μM]**	**Spiked plasma Measured conc. [μM] mean ± SD**	**Recovery [%]**	**Within-run precision (CV%) (n = 5)**	**Between-run precision (CV%) (n = 10)**
		0.108	0.280±0.014	101.8	7.4	5.2
Cortisol	0.171±0.003	0.20	0.364±0.020	98.4	5.5	5.4
		0.33	0.486±0.023	94.8	6.5	4.6

LOD, limit of detection; LOQ, limit of quantification; CV, coefficient of variation, R^2, regression coefficient; conc., concentration; SD, standard deviation.

Table S2. Baseline plasma cortisol association with patient- and cancer-related features.

Parameter	Mean cortisol±SD [ng/ml] or correlation coefficient	*P* Value
Age	*r* = 0.07	0.567
Sex, F vs. M	80.9±29.7 vs. 84.2±31.1	0.649
BMI	*ρ* = -0.14	0.236
ASAPS, 1 vs. 2 vs. 3	77.9±25.3 vs. 83.4±26.7 vs. 88.5±49.6	0.659
CCS	*ρ* = -0.02	0.839
TNM, 0 vs. I vs. II vs. III vs IV	85.5±35.7 vs. 101.3±56.5 vs. 80.5±21.4 vs. 80.3±30.2 vs. 87.4±37.1	0.609
T, Tis vs. 1 vs. 2 vs. 3 vs. 4	85.5± 35.7 vs. 91.9 vs. 93.9±49.8 vs. 80.9±27.5 vs. 80.8±24.7	0.823
N, 0 vs. 1 vs. 2	84.3±30.8 vs. 80.3±36.1 vs. 82.6±23.4	0.893
M, 0 vs. 1	82.5±30.1 vs. 87.4 vs. 37.1	0.731
G, 0 vs. 1 vs. 2 vs. 3 vs. 4	94.6±51.9 vs. 80.0±27.3 vs. 87.9±22.2 vs. 91.9	0.551
Anatomical site, RC vs. LC vs. R	87.4±29.3 vs. 81.5±30.4 vs. 80.7±31.7	0.708

Analysis of baseline cortisol was conducted for all 76 patients. SD, standard deviation; F, females; M, males; BMI, body mass index; ASAPS, the American Society of Anesthesiologists physical status classification system; CCS, the Charlson comorbidity score; TNM, the tumor-node-metastasis cancer staging system; G, histopathological grade; RC, right colon; LC, left colon; R, rectum; *r*; Pearson correlation coefficient; *ρ*, Spearman rank correlation.

Table S3. Impact of patient-related features on mean cortisol concentration during perioperative period

Factor	Group	Cortisol [ng/ml], geometric mean (95% CI)			
		**Preoperative**	**At 8 hours**	**At 24 hours**	**At 72 hours**
**Sex**	Females	74.3 (64-86.3)	128.3 (96.7-170.3)	108.4 (86.9-135.3)	86.1 (72.1-102.8)
	Males	83.3 (74.6-93.1)	98.8 (80.5-121.1)	88.8 (76.6-103)	75.2 (64.4-87.7)
**Age**	<75 yrs.	81.7 (74.6-89.5)	110.8 (92.5-132.7)	92.9 (80.6-107)	79 (68.9-90.6)
	≥75 yrs.	76.4 (62.2-93.8)	104.1 (72.4-149.6)	100.9 (78.4-129.9)	78.7 (62.3-99.5)
**BMI**	<25	82.2 (70.4-96)	98.3 (69.6-138.8)	89.3 (73.3-108.7)	81.1 (66.2-99.2)
	≥25	78.5 (70.3-87.6)	115.8 (98.2-136.5)	99.7 (84.7-117.2)	77.6 (67-89.8)
**ASAPS**	ASAPS=1	77.2 (62.4-95.5)	149.5 (124.2-179.8)	101 (81.2-124.6)	77.4 (57-105.1)
	ASAPS=2	81.3 (74.3-88.9)	97.8 (82.9-115.4)	93.5 (80.3-108.9)	82.5 (72.5-93.9)
	ASAPS=3	78 (52.2-116.5)	110.9 (48.3-254.2)	96.5 (59.6-156.1)	67.9 (43.8-105)

CI, confidence interval; yrs. years; BMI, body mas index; ASAPS, the American Society of Anesthesiologists physical status classification system.

Table S4. Impact of surgery-related features on mean cortisol concentration during perioperative period

Factor	Group	Cortisol [ng/ml], geometric mean (95% CI)			
		Preoperative	At 8 hours	At 24 hours	At 72 hours
Surgery type	Open	81.4 (74.3-89.3)	120.7 (94.1-155)	114 (97.9-132.6)	83.4 (72.7-95.7)
	Robotic	78.1 (66-92.3)	94.9 (77.5-116.2)	76.3 (64.1-90.9)	73.6 (60-90.3)
EBL	<150 ml.	74.1 (65.2-84.2)	101.9 (82.7-125.6)	83.7 (71.2-98.4)	74.5 (62.4-88.9)
	≥150 ml	85.6 (75.8-96.7)	114.9 (88.8-148.8)	107.5 (89.8-128.6)	83.2 (70.9-97.5)
Excised LN	<15	75.3 (66.2-85.5)	102 (76.8-135.5)	90.6 (75.4-108.9)	72.2 (60.5-86.2)
	≥15	85.4 (75.6-96.4)	116.3 (98.9-136.7)	101 (85.3-119.7)	87.1 (75.1-101.1)
LoS	<175 min	80.1 (71.1-90.1)	109.6 (90.8-132.3)	106.8 (92.8-122.8)	89.2 (78.2-101.8)
	≥175 min	79.7 (69.4-91.6)	107 (78.3-146.2)	81.2 (65.3-100.9)	66.2 (54.2-80.8)

CI, confidence interval; EBL, estimated blood loss; LN, lymph nodes; LoS, length of surgery.
